# Diagnostic value of super-resolution ultrasound imaging in differentiating benign and malignant BI-RADS-4 breast lesions

**DOI:** 10.3389/fonc.2025.1662492

**Published:** 2025-10-06

**Authors:** Xueqin Hou, Zhiming Li, Yibin Liu, Junxi Gao, Tao Song

**Affiliations:** Department of Abdominal Ultrasound, The First Affiliated Hospital of Xinjiang Medical University, Xinjiang Key Laboratory of Ultrasound Medicine, Urumqi, China

**Keywords:** SRUS, BI-RADS 4 category, breast lesions, nomogram, prediction model

## Abstract

**Objective:**

Benign and malignant breast tumors exhibit distinct microvascular morphological patterns and spatial distribution characteristics; however, current clinical imaging modalities cannot comprehensively assess their microvascular network architecture. Super-resolution ultrasound (SRUS) imaging addresses this critical gap by providing super-resolved visualization of microvascular topology. This study aimed to evaluate the utility of SRUS imaging in visualizing breast lesion microvasculature and establishing diagnostic models for BI-RADS 4 masses.

**Methods:**

A total of 120 breast lesions from 117 patients with conventional ultrasound-confirmed BI-RADS 4 lesions were prospectively enrolled in this study between July 2024 and January 2025. Preoperative conventional ultrasound and SRUS examinations were performed on all included patients. Based on pathological findings, the lesions were categorized as benign (n = 85) or malignant (n = 35). The benign group was further stratified into hypovascular (n = 42) and hypervascular (n = 43) subgroups based on the SRUS enhancement levels. Univariate analysis was performed to screen SRUS parameters, and variables with *P* < 0.05 were incorporated into multivariate logistic regression models to construct nomogram-based predictive models and validated using ROC analysis.

**Results:**

Max vel (OR = 1.848, 95% CI: 1.205–3.122), curvature (A/E) (OR = 2.162, 95% CI: 1.981–2.323), and complexity level (OR = 1.772, 95% CI: 1.608–1.942) independently predicted malignancy (all *P* < 0.05). Curvature (A/E) and complexity level were also independent markers for distinguishing malignant lesions from hypervascular benign lesions (curvature (A/E): OR = 1.808, 95% CI: 1.612–1.987; complexity level: OR = 1.952, 95% CI: 1.804–2.181; both *P* < 0.05). The nomogram prediction models demonstrated high diagnostic efficacy, with AUC values of 0.899 (95% CI: 0.844–0.953) (sensitivity = 94.3%, specificity = 76.5%) for benign compared to 0.816 (95% CI: 0.721–0.910 (sensitivity = 65.1%, specificity = 88.6%) for malignant differentiation. The Hosmer-Lemeshow goodness-of-fit test indicated adequate model fit (all *P* > 0.05). The nomogram prediction model demonstrated superior net benefit in predicting breast cancer compared to alternative strategies.

**Conclusion:**

SRUS enables microvascular characterization of breast lesions, with validated nomograms demonstrating high diagnostic accuracy for early cancer detection.

## 1 Introduction

Breast cancer is the most prevalent malignancy among women worldwide, with the highest incidence and mortality rates; this trend underscores the urgent need for precise early diagnosis, a persistent challenge in modern clinical medicine (1). Ultrasonography is widely utilized for screening breast nodules due to its non-ionizing nature, user-friendly operation, and cost-effectiveness (2). However, conventional ultrasound imaging is constrained by the diffraction limit (~200 μm), which impairs the visualization of critical malignant features, such as microcalcifications and neovascularization. This limitation contributes to a diagnostic uncertainty in 10%–30% of cases (3).

Super-Resolution Ultrasound (SRUS) imaging is an advanced tracking technique that employs ultrafast plane-wave ultrasound imaging to achieve microbubble localization, tracking, and trajectory reconstruction. By transcending the spatial resolution limitations (typically > 200 μm) inherent to conventional Doppler-based hemodynamic imaging, this platform achieves microvascular flow mapping with quantified spatial precision of 25–50 μm. Such technical advancements establish SRUS as a transformative tool for both mechanistic investigations of microcirculatory pathophysiology and clinical decision-making in vasculopathic disorders, particularly for early-stage microangiopathy detection (4). The technology has been successfully applied in both preclinical animal models and clinical trials, including lymph node characterization (5), tumor evaluation in the brain and liver (6, 7), ocular vascularization analysis (8), cerebral microperfusion studies (9), testicular lesion characterization (10), and microcirculatory assessment in acute kidney injury (11). This study aimed to evaluate the clinical utility of SRUS for breast lesion characterization, offering a potential breakthrough in precision diagnostics.

## 2 Materials and methods

### 2.1 Patients

A prospective cohort of 117 patients with breast masses detected via conventional ultrasound was enrolled between July 2024 and January 2025. The patients’ ages ranged from 20 to 83 years (mean: 45.17 ± 11.75 years). A total of 120 lesions were included, with maximum diameters ranging from 3.9 to 55.9 mm (mean: 17.70 ± 11.58 mm; median: 15 mm). All lesions were classified as BI-RADS category 4 on conventional ultrasound and subsequently categorized as benign (n = 85) or malignant (n = 35) based on histopathological results. Benign lesions were further stratified into hypervascular (n = 43) and hypovascular (n = 42) subgroups. This study was approved by the ethics committee of the First Affiliated Hospital of Xinjiang Medical University (approval no.: K202310-02), and all experiments were performed in accordance with relevant guidelines and regulations. Written informed consent was obtained from all participants, and all procedures were performed in accordance with the Helsinki Declaration.

The study’s inclusion criteria were as follows: (1) Complete pre-biopsy/preoperative conventional ultrasound and SRUS imaging data; and (2) availability of definitive histopathological and clinical records. The exclusion criteria were as follows: (1) Pregnancy or lactation; (2) contraindications to ultrasound contrast agents; (3) prior tumor-related therapies; (4) history of concurrent malignancies; and (5) loss to follow-up or incomplete imaging data.

### 2.2 Equipment and contrast agent

Ultrasound examinations were performed using the ULTIMUS 9E (VINNO) system equipped with a U5-15 linear array transducer (6–12 MHz, breast imaging preset). SRUS imaging and conventional ultrasound parameters were standardized as follows: contrast auto-capture interval of 120 s, SRUS acquisition duration of 15 s, low contrast agent type, and acoustic power set at 9%. The contrast agent used was SonoVue^®^ (Bracco, Milan, Italy).

### 2.3 Ultrasound protocol

#### 2.3.1 Conventional ultrasound

The patients were positioned supine with both arms elevated to fully expose the breast and axilla. Grayscale imaging captured representative cross-sections, maximum diameter planes, and orthogonal views, while color Doppler flow imaging (CDFI) and super microvascular flow (SMF) modes were optimized to visualize the most vascularized cross-sections.

#### 2.3.2 SRUS imaging

The largest diameter or most vascularized plane was selected for SRUS. A freeze-dried SonoVue^®^ powder suspension was prepared by dissolving it in 5 mL of normal saline, forming a milky white microbubble solution. A 2.5-mL aliquot of this suspension was then injected into a peripheral vein at a constant rate, followed by a 5-mL saline flush. Following the injection of the contrast agent, a 120-second imaging sequence was immediately commenced in the real-time dual contrast-enhanced ultrasound (CEUS) mode. Once microbubbles entered the tumor, the “URM (ultra-resolution microscopy imaging) Collection” button was pressed to capture 15 s of imaging data.

### 2.4 Stratification of benign lesions

Due to the inherent technical limitations of CDFI and SMF in detecting low-velocity and fine vascular signals, this study classified benign lesions into hypervascular and hypovascular subgroups based on CEUS enhancement patterns. Hypervascular lesions were those exhibiting predominantly high enhancement on CEUS, characterized by marked contrast agent filling with intensity greater than that of the surrounding normal breast tissue. Hypovascular lesions showed predominantly low or iso-enhancement, where the degree of contrast agent filling was similar to or lower than that of the surrounding tissue.

### 2.5 Image analysis

SRUS parameters were independently measured by two senior sonographers who were blinded to the patients’ clinical history and prior ultrasound results. The final SRUS values were derived as the average of both assessments. Conventional ultrasound recorded the characteristics of the lesion, including the longest diameter, growth orientation (parallel or non-parallel to the skin), margin irregularities (presence of burrs), calcifications, and the presence of a hyperechoic halo. CDFI and SMF were then applied to assess tumor vascularization. Vascularity was classified using the Alder semi-quantitative blood flow grading method: grade 0–I (low vascularity) and grade II–III (high vascularity) (12).

SRUS image analysis was conducted using the “URM Video” mode, with the region of interest (ROI) encompassing the lesion and surrounding tissue. After defining the ROI, the “Analysis” function was activated, and measurements were performed using density analysis, DV analysis, curvature A/E, and perfusion index tools. Lesion boundaries were manually traced to extract structural parameters (vessel ratio, complexity level, density [max, min, mean], and curvature A/E), hemodynamic parameters (microvascular velocity [max, min, mean]), and comprehensive parameters (perfusion index: velocity × density).

### 2.6 Definition of SRUS parameters

The vessel ratio was defined as the area of vascular perfusion within the region of interest (ROI) divided by the total area of the ROI.


Vessel ratio = Area of vascular perfusion within the ROI/Total area of the ROI.

This parameter reflects the richness of blood supply in the region of interest and may serve as a potential indicator for differential diagnosis and evaluation of disease progression.

The complexity level serves as a metric for describing the degree of structural intricacy within an image. It quantifies complexity as the ratio of variation in graphical detail to changes in measurement scale—a higher value indicates a greater degree of complexity. Based on contrast-enhanced microvascular density maps, the fractal dimension within ROI was calculated, reflecting the morphological complexity of the vascular network in the area.

Vessel density represents the number of microbubbles passing through that point during the acquisition period (typically 10 s). A higher microbubble count corresponds to a greater density value.

Curvature (A/E) was used to describe the tortuosity of the vascular path. A higher value indicated a greater degree of vessel bending.

Vessel velocity was defined as the displacement of microbubbles per unit time within the region of interest (ROI). The perfusion index was calculated as the product of mean velocity and vessel density ratio within the ROI.


Perfusion index = Mean Velocity within the ROI × Vessel Density Ratio in the ROI.

Velocity referred to the speed of blood flow, while density represented the quantity of red blood cells. Their product provided a more accurate reflection of tissue perfusion.

### 2.7 Statistical analysis

Data analysis was performed using R software (version 4.2.1; R Foundation for Statistical Computing, Vienna, Austria). The normality of continuous variables was assessed using the Shapiro-Wilk test. Normally distributed data were expressed as mean ± standard deviation (x ± s) and analyzed using independent-samples t-tests for two-group comparisons, one-way ANOVA for multi-group comparisons, and least significant difference (LSD) *post hoc* tests for pairwise analysis. Non-normally distributed data were reported as median (Q1, Q3) and analyzed using the Mann-Whitney U test (two-group comparisons) and the Kruskal-Wallis test (multi-group comparisons). Categorical variables were presented as frequencies (%) and analyzed using Pearson’s chi-square test or Fisher’s exact test, as appropriate. A two-tailed *P*-value <0.05 was considered statistically significant. Variables with *P* < 0.05 in univariate analyses were incorporated into multivariate logistic regression models, with the condition parameter set as the estimated likelihood ratio test, to construct a nomogram-based prediction model. The predictive performance of the model was assessed using the area under the receiver operating characteristic (ROC) curve (AUC). Model goodness-of-fit was evaluated through the Hosmer-Lemeshow test, with calibration curves generated to demonstrate prediction reliability. Additionally, decision curve analysis (DCA) was employed to quantify the net benefit of the model in stratifying breast cancer incidence risk across clinically relevant probability thresholds.

## 3 Results

### 3.1 Comparison of clinical characteristics and conventional ultrasound features between benign and malignant breast lesions

Compared with patients in the benign group, those in the malignant group were significantly older and had larger maximum lesion diameters. Malignant lesions predominantly exhibited marked hypoechogenicity, internal heterogeneity, spiculated or crab claw-like margins, and intralesional microcalcifications, with significantly higher vascularity (all *P* < 0.05). No significant differences were observed in lesion location, orientation, or growth direction between both groups (*P* > 0.05, **Table 1**).

**Table 1:** Comparison of clinical characteristics and conventional ultrasound features between benign and malignant breast nodule groups.

Variables	Benign group (n = 85)	Malignant group (n = 35)	*χ²/Z*	*P*
Age (years)	42.00 (35.00–49.00)	50.00 (44.50–59.00)	4.639	<0.001
Position			0.997	0.318
Left	35 (41.18)	11 (31.43)		
Right	50 (58.82)	24 (68.57)		
Anatomical location			0.803	0.849
Upper inner quadrant	26 (30.59)	11 (31.43)		
Lower inner quadrant	7 (8.24)	2 (5.71)		
Upper outer quadrant	41 (48.24)	19 (54.29)		
Lower outer quadrant	11 (12.94)	3 (8.57)		
Maximum diameter (mm)	14.00 (8.30–18.00)	23.50 (11.75–36.05)	3.669	<0.001
Echogenicity			7.571	0.023
Marked hypoechoic	36 (42.36)	17 (48.57)		
Hypoechoic	47 (55.29)	14 (40.00)		
Others	2 (2.35)	4 (11.43)		
Hyperechoic halo			0.252	0.616
Without	69 (81.18)	27 (77.14)		
With	16 (18.82)	8 (22.86)		
Shape			6.303	0.012
Regular	22 (25.88)	2 (5.71)		
Irregular	63 (74.12)	33 (94.29)		
Margin			2.353	0.125
Circumscribed	29 (34.12)	7 (20.00)		
Indistinct	56 (65.88)	28 (80.00)		
Internal echotexture uniformity			8.056	0.005
Homogeneity	46 (54.12)	9 (25.71)		
Heterogeneity	39 (45.88)	26 (74.29)		
Growth orientation			2.269	0.132
Parallel, wider than tall	71 (83.53)	25 (71.43)		
Vertical, taller than wide	14 (16.47)	10 (28.57)		
Blur margin			19.274	<0.001
Without	72 (84.71)	16 (45.71)		
With	13 (15.29)	19 (54.29)		
Calcification			41.572	<0.001
Without	80 (94.12)	15 (42.86)		
Micro	3 (3.53)	18 (51.43)		
Coarse	2 (2.35)	2 (5.71)		
CDFI			27.576	<0.001
Grade 0–I	79 (92.94)	18 (51.43)		
Grade II–III	6 (7.06)	17 (48.57)		
SMF			31.775	<0.001
Grade 0–I	68 (80.00)	9 (25.71)		
Grade II–III	17 (20.00)	26 (74.29)		

### 3.2 Comparison of SRUS parameters between benign and malignant breast lesions

Significant differences in SRUS parameters were observed between benign and malignant lesions, including vessel ratio, complexity level, maximum/mean density, maximum/mean velocity, curvature (A/E), and perfusion index, with malignant lesions exhibiting higher values (*P* < 0.05). However, minimum microvascular density and velocity showed no significant intergroup differences (*P* > 0.05, **Table 2**).

**Table 2:** Comparison of SRUS parameters between the benign and malignant breast nodule groups.

Variables	Benign group (n = 85)	Malignant group (n = 35)	*t/Z*	*P*
Vessel ratio (%)	11.48 (5.00, 23.37)	33.24 (26.98, 45.41)	5.52	<0.001
Complexity level	1.36 (1.17, 1.48)	1.62 (1.55, 1.71)	6.632	<0.001
Density				
Max	18.32 ± 8.69	22.13 ± 6.79	2.317	0.022
Min	0.09 (0.07, 0.12)	0.11 (0.08, 0.12)	1.331	0.183
Mean	5.03 ± 2.51	7.02 ± 1.61	4.323	<0.001
Velocity (mm/s)				
Max	20.48 (17.68, 23.00)	23.00 (21.65, 25.18)	4.579	<0.001
Min	0.09 (0.09, 0.10)	0.09 (0.09, 0.10)	0.118	0.907
Mean	5.58 (3.97, 7.09)	8.27 (6.18, 10.47)	4.657	<0.001
Curvature (A/E)	1.12 (1.07, 1.18)	1.30 (1.19, 1.54)	5.234	<0.001
Perfusion index	1.14 (0.34, 2.40)	3.55 (2.48, 4.38)	5.301	<0.001

### 3.3 Comparison of SRUS parameters across three subgroups and *post hoc* analyses

**Figure 1:**
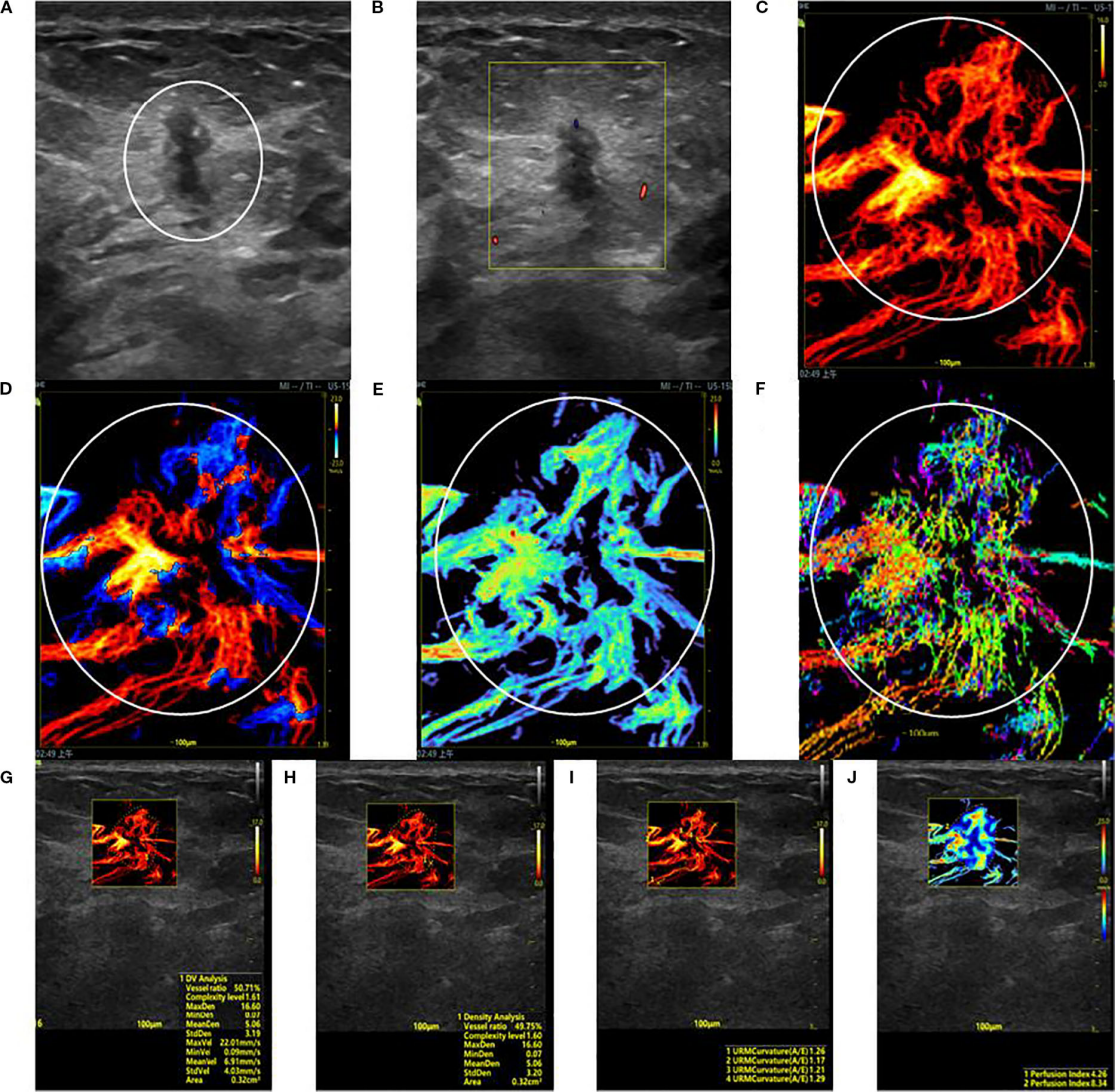
SRUS imaging of a malignant breast lesion: **(A)** Grayscale ultrasound findings suggest the presence of a hypoechoic mass (3.9 × 4.8 mm) with longitudinal orientation, ill-defined margins, irregular shape, heterogeneous internal echogenicity, angular edges, and multiple microcalcifications. **(B)** SMF image with Alder grade I classification. Density map **(C)**, Orientation map **(D)**, Velocity map **(E)**, and Angle map **(F)**, with the lesion region delineated by circular markers based on SRUS images and associated dynamic sequences. **(G–J)** SRUS parameter quantification panels: density ratio (49.75%), complexity level (1.60), maximum density (16.60), minimum density (0.07), mean density (5.06), maximum velocity (22.01 mm/s), minimum velocity (0.09 mm/s), mean velocity (6.91 mm/s), curvature A/E (1.26), and Perfusion Index (4.26).

**Figure 2:**
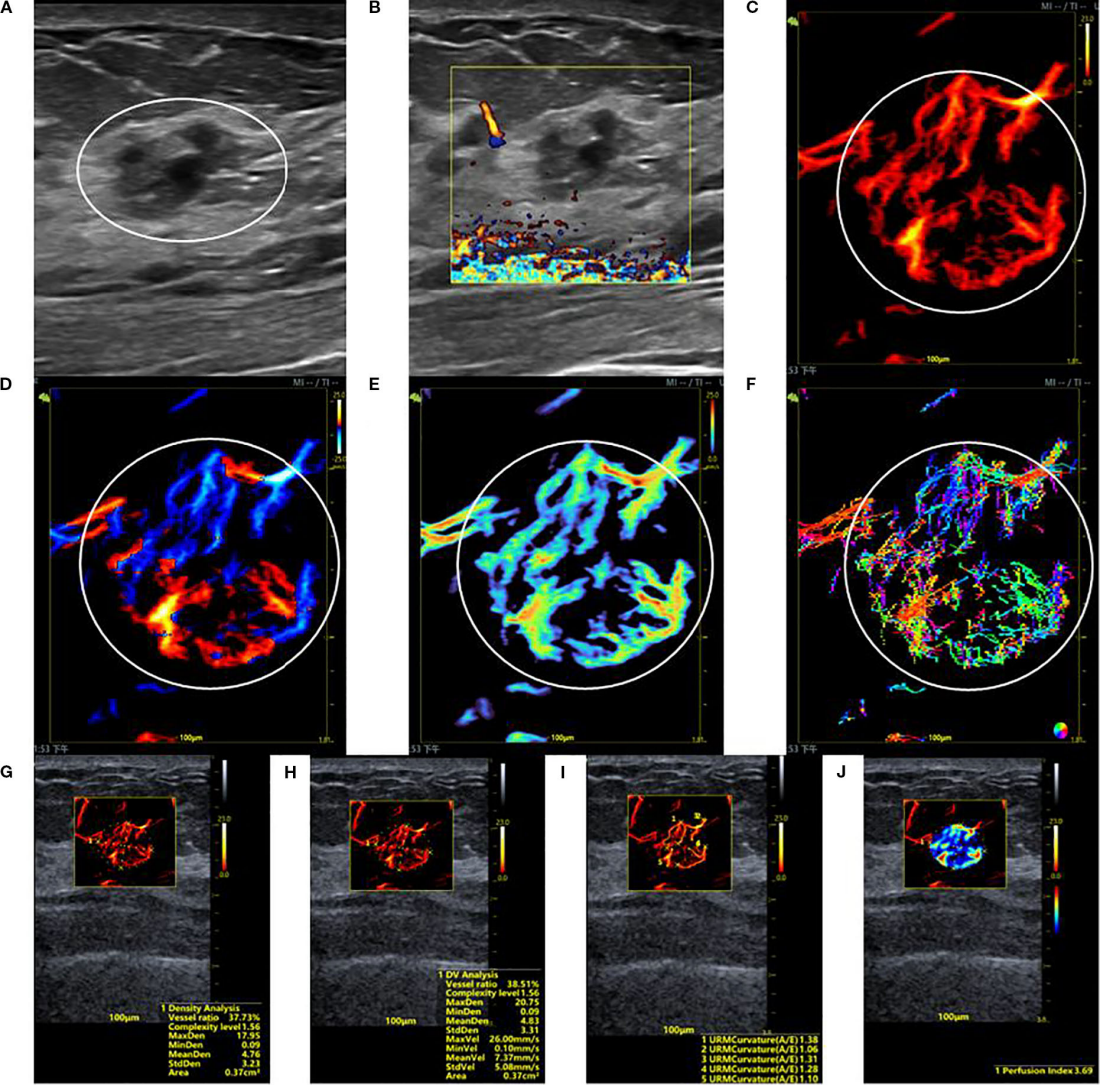
SRUS imaging of a hypervascular benign breast lesion: **(A)** Grayscale ultrasound findings suggest the presence of a hypoechoic mass (15.2 × 10.0 mm) with parallel orientation, ill-defined margins, irregular shape, and heterogeneous internal echogenicity. **(B)** SMF image classified as Alder grade 0. Density map **(C)**, Orientation map **(D)**, Velocity map **(E)**, and Angle map **(F)**, with the lesion region delineated by circular markers based on SRUS images and associated dynamic sequences. **(G–J)** SRUS parameter quantification panels: density ratio (37.73%), complexity level (1.56), maximum density (17.95), minimum density (0.09), mean density (4.76), maximum velocity (26.00 mm/s), minimum velocity (0.10 mm/s), mean velocity (7.37 mm/s), curvature A/E (1.28), and Perfusion Index (3.69).

**Figure 3:**
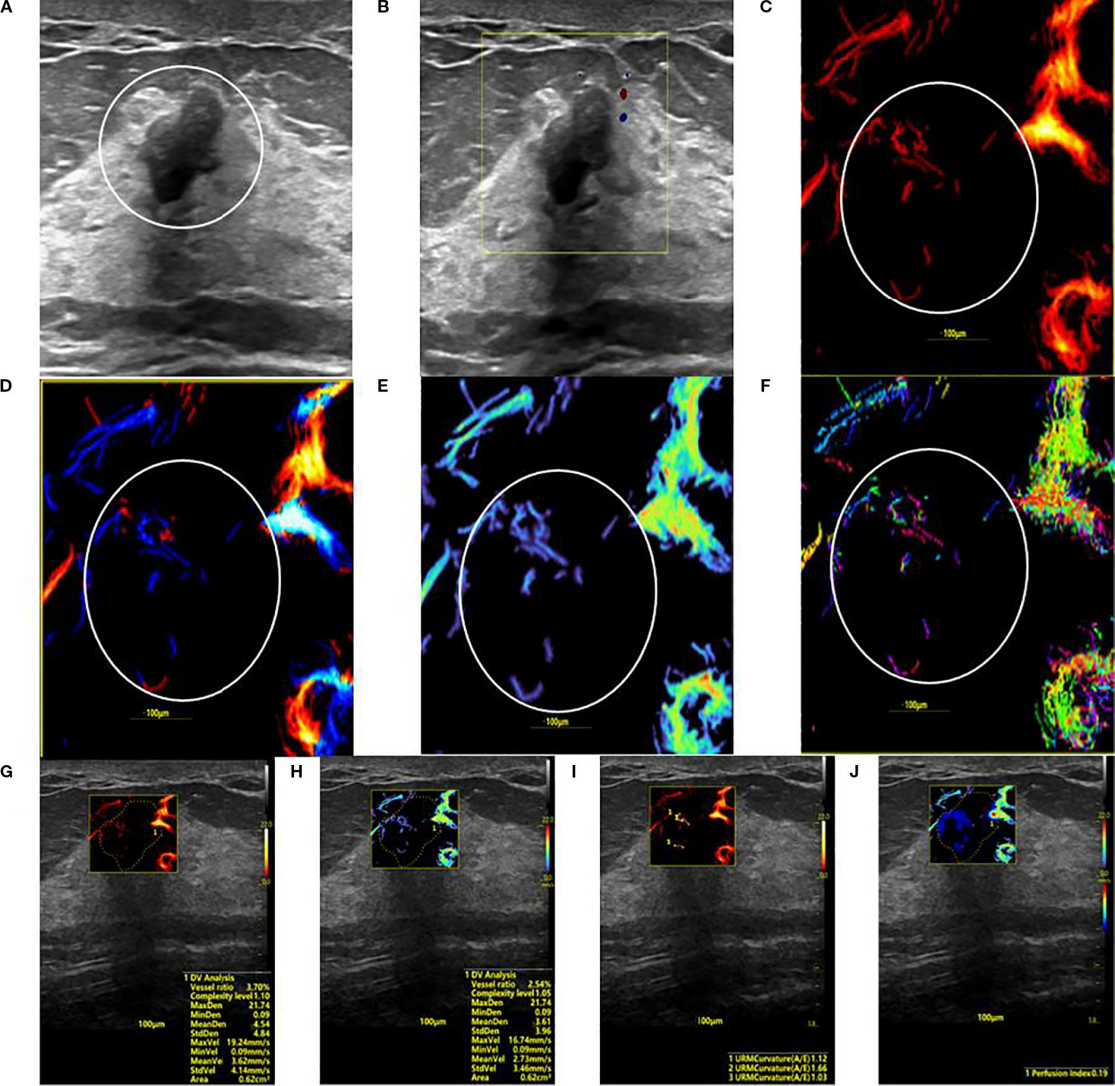
SRUS imaging of a hypovascular benign breast lesion: **(A)** Grayscale ultrasound findings suggest the presence of a hypoechoic mass (8.3 × 5.1 mm) with parallel orientation, well-defined margins, irregular shape, and heterogeneous internal echogenicity. **(B)** SMF image categorized as Alder grade 0. Density map **(C)**, Orientation map **(D)**, Velocity map **(E)**, and Angle map **(F)**, with the lesion region delineated by circular markers based on SRUS images and associated dynamic sequences. **(G–J)** SRUS parameter quantification panels: density ratio (2.79%), complexity level (1.05), maximum density (21.74), minimum density (0.09), mean density (3.61), maximum velocity (16.74 mm/s), minimum velocity (0.09 mm/s), mean velocity (2.73 mm/s), curvature A/E (1.12), and Perfusion Index (0.19).

Among the three groups (malignant, hypervascular benign, and hypovascular benign), statistically significant differences were found in vessel ratio, complexity level, maximum/minimum/mean density, maximum/mean velocity, curvature (A/E), and perfusion index (all *P* < 0.05). *Post hoc* pairwise comparisons revealed a hierarchical pattern: malignant > hypervascular benign > hypovascular benign for all significant parameters (all *P* < 0.05). The minimum microvascular velocity did not differ significantly among groups (P > 0.05). Furthermore, the malignant group exhibited significantly higher values than the hypervascular benign group in microvascular density ratio, vascular complexity, maximum/mean velocity, curvature (A/E), and perfusion index (all *P* < 0.05, **Table 3**, **Figures 1**–**3**).

**Table 3:** Comparison of SRUS parameters among the three groups.

Variables	Hypovasculargroup (n=42)	Hypervasculargroup (n=43)	Malignantgroup(n=35)	*F/Z*	*P*
Vessel ratio (%)	6.69 (2.27, 10.24)^a^	20.64 (13.57, 36.83)^b^	33.24 (26.98, 45.41)^c^	7.619	<0.001
Complexity level	1.18 (1.01, 1.34)^a^	1.46 (1.38, 1.56)^b^	1.62 (1.55, 1.71)^c^	7.984	<0.001
Density					
Max	14.27 ± 7.24^a^	22.27 ± 8.21	22.13 ± 6.79^c^	3.92	<0.001
Min	0.08 (0.06, 0.10)^a^	0.10 (0.08, 0.13)	0.11 (0.08, 0.12)^c^	3.118	0.008
Mean	3.82 ± 1.83^a^	6.22 ± 2.54	7.02 ± 1.61^c^	5.094	<0.001
Velocity (mm/s)					
Max	19.20 (15.48, 20.62)^a^	21.65 (20.20, 24.00)^b^	23.00 (21.65, 25.18)^c^	6.026	<0.001
Min	0.09 (0.09, 0.10)	0.09 (0.09, 0.10)	0.09 (0.09, 0.10)	2.176	0.094
Mean	4.40 (3.18, 5.83)^a^	6.41 (4.96, 8.04)^b^	8.27 (6.18, 10.47)^c^	6.288	<0.001
Curvature (A/E)	1.10 (1.05, 1.15)^a^	1.13 (1.08, 1.23)^b^	1.30 (1.19, 1.54)^c^	5.636	<0.001
Perfusion index	0.50 (0.10, 0.84)^a^	2.01 (1.21, 3.92)^b^	3.55 (2.48, 4.38)^c^	7.413	<0.001

^a^Hypovascular benign subgroup vs. hypervascular benign subgroup, *P* < 0.05; ^b^Hypervascular benign subgroup vs. malignant group, *P* < 0.05; ^c^Hypovascular benign subgroup vs. malignant group, *P* < 0.05.

### 3.4 Model construction

**Figure 4:**
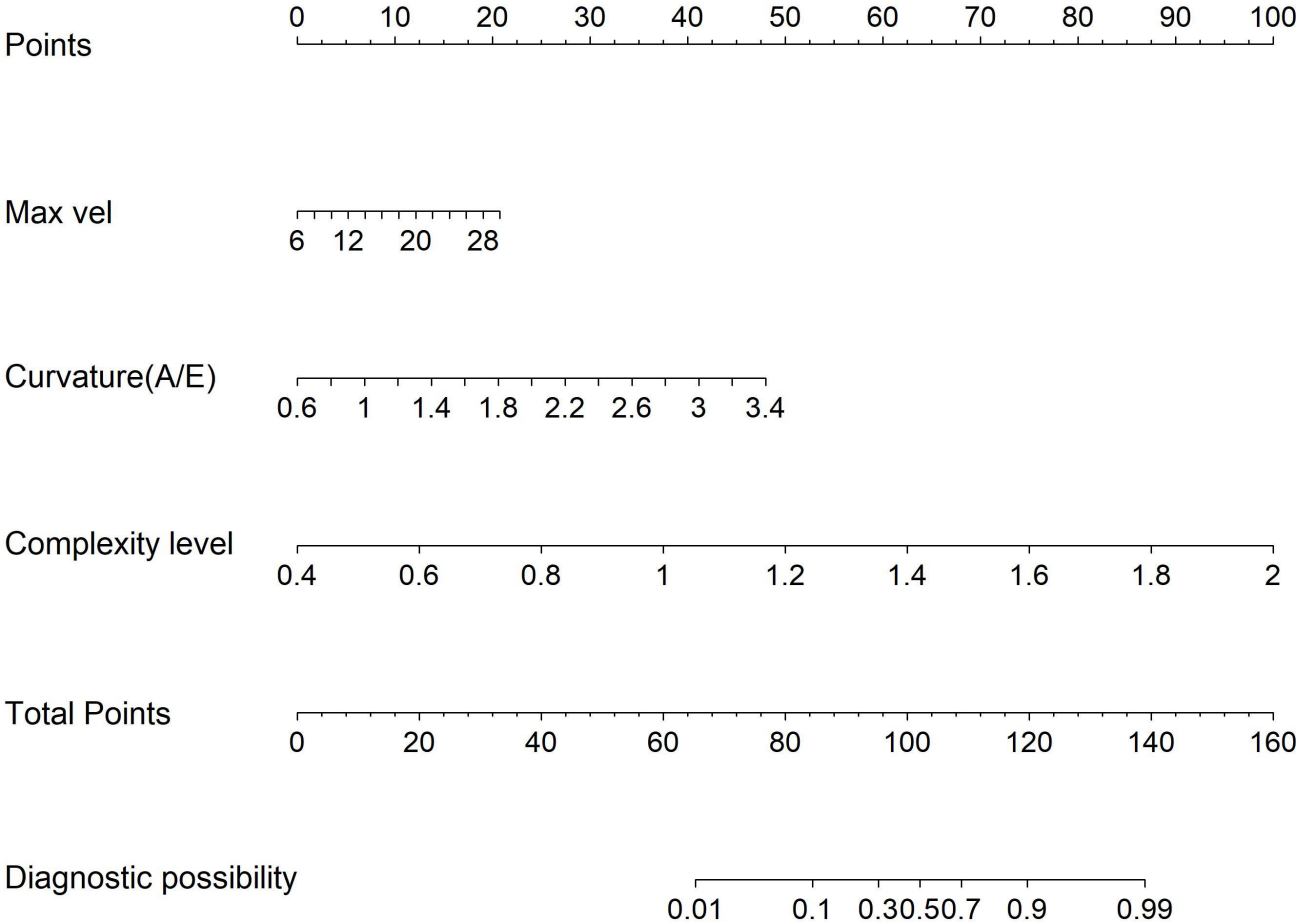
Model 1: Nomogram for discriminating benign from malignant BI-RADS category 4 breast lesions using URM parameters.

**Figure 5:**
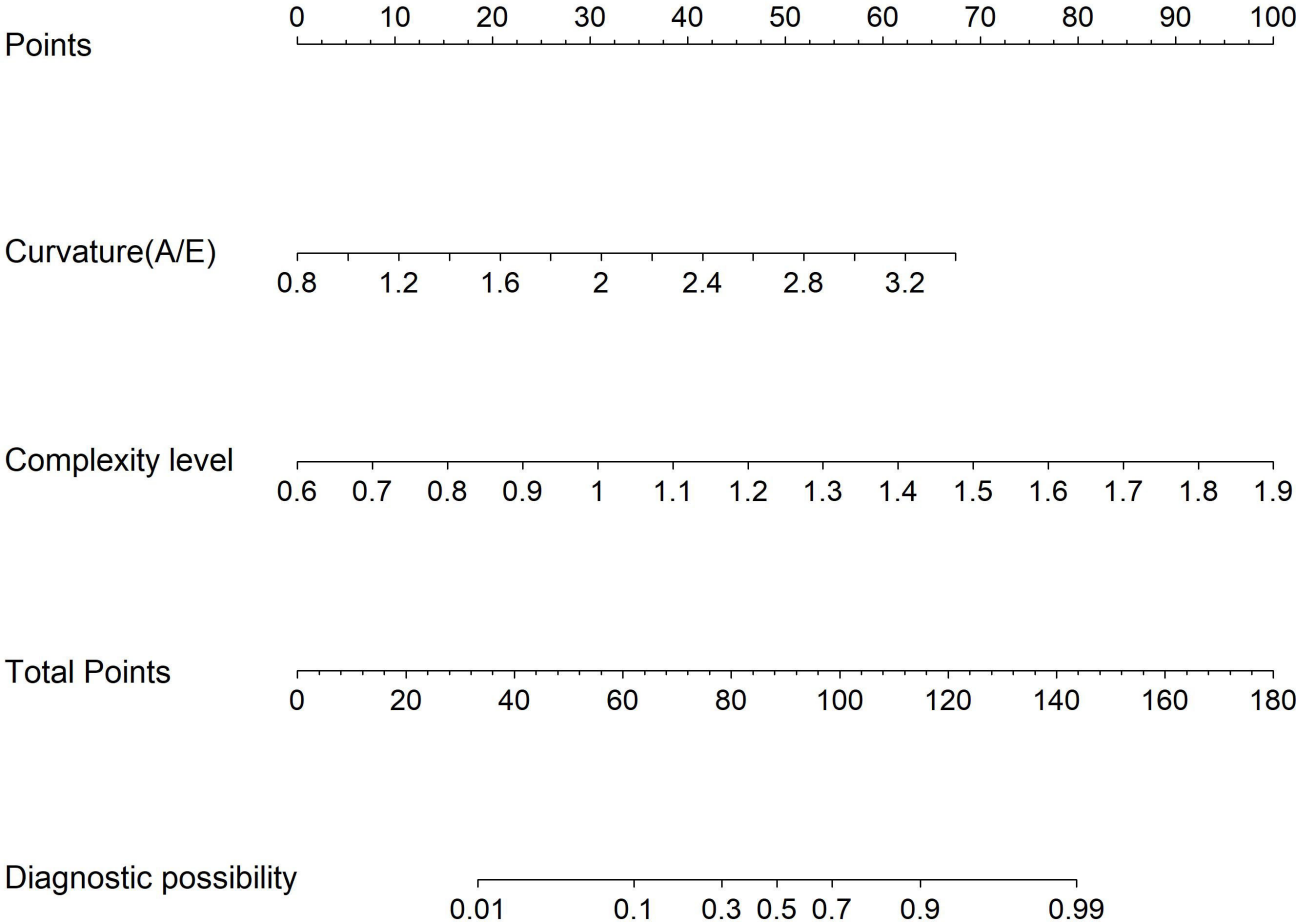
Model 2: Nomogram for differentiating hypervascular benign and malignant BI-RADS category 4 breast lesions based on URM parameters.

Multivariate logistic regression analysis incorporated variables with statistically significant differences. Max vel, curvature (A/E), and complexity level emerged as independent predictors for distinguishing benign from malignant BI-RADS category 4 lesions: OR = 1.848 (95% CI: 1.205–3.122), OR = 2.162 (95% CI: 1.981–2.323), and OR = 1.772 (95% CI: 1.608–1.942; all *P* < 0.05, **Table 4**). Additionally, curvature (A/E) and complexity level independently discriminated hypervascular benign from malignant BI-RADS 4 lesions: OR = 1.808 (95% CI: 1.612–1.987), OR = 1.952 (95% CI: 1.804–2.181; both *P* < 0.05, **Table 5**). Separate nomogram prediction models were developed based on these variables, with breast cancer occurrence as the endpoint (**Figures 4**, **5**). The model assigned individual scores to each risk factor, and the sum of these scores corresponded to the predicted probability of breast cancer, where higher totals indicate an elevated malignancy risk.

**Table 4:** Multivariable logistic regression analysis of SRUS parameters in differentiating benign from malignant breast nodules.

Variables	*β*	*SE*	*Z*	*OR(95%CI)*	*P*
Max vel	0.614	0.239	2.568	1.848 (1.205, 3.122)	0.010
Curvature (A/E)	0.771	0.368	2.095	2.162 (1.981, 2.323)	0.036
Complexity level	0.572	0.245	2.335	1.772 (1.608, 1.942)	0.020

**Table 5:** Multivariable logistic regression analysis of SRUS parameters for differentiating hypervascular benign from malignant breast lesions.

Variables	*β*	*SE*	*Z*	*OR(95%CI)*	*P*
Curvature (A/E)	0.592	0.245	2.416	1.808 (1.612, 1.987)	0.016
Complexity level	0.669	0.305	2.193	1.952 (1.804, 2.181)	0.028

### 3.5 Model validation

**Figure 6:**
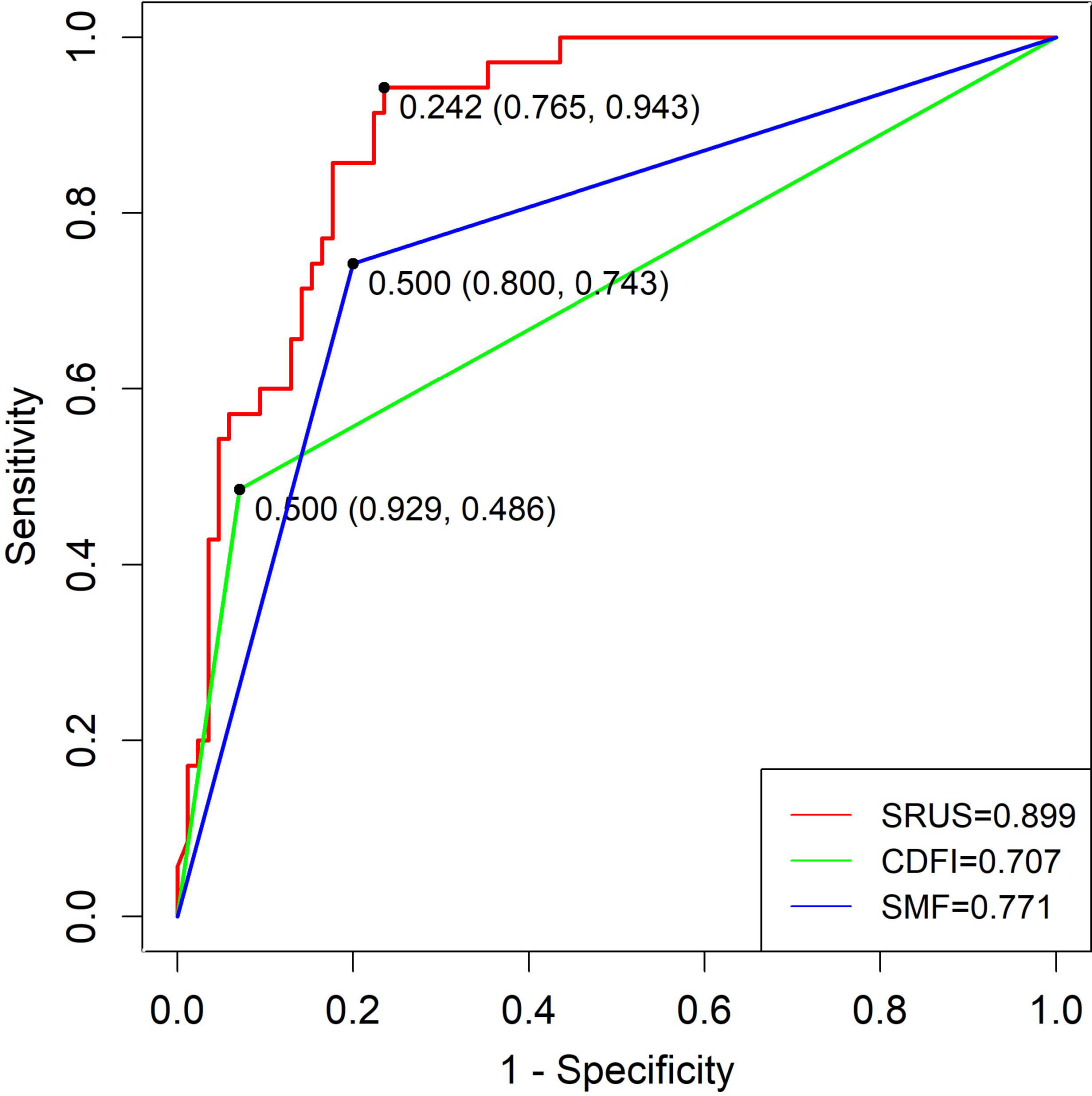
ROC curves of SRUS, CDFI, and SMF for the differentiation of malignant and benign breast nodules.

The SRUS-based nomogram for discriminating benign from malignant lesions demonstrated an AUC of 0.899 (95% CI: 0.844–0.953), with a sensitivity of 94.3% and a specificity of 76.5% at the optimal cutoff of 0.242. By comparison, CDFI and SMF showed lower AUC values (0.707 and 0.771, respectively), with sensitivities of 68.6% and 80.0%, and specificities of 72.9% and 65.9%, respectively (**Figure 6**).

**Figure 7:**
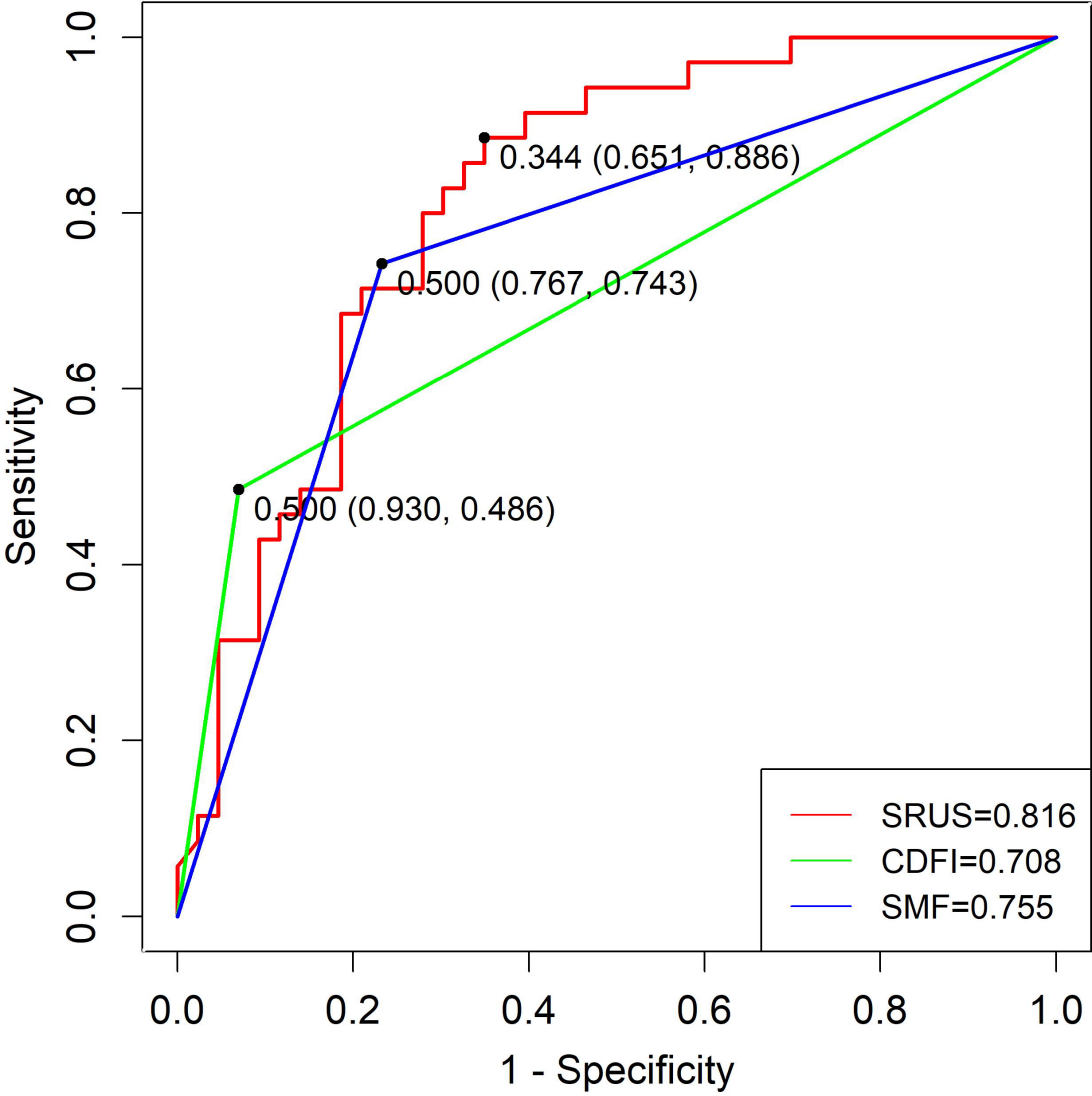
ROC curves of SRUS, CDFI, and SMF for the discrimination between malignant and hypervascular benign solid breast nodules.

Regarding distinguishing malignant from hypervascular benign lesions, the SRUS model achieved an AUC of 0.816 (95% CI: 0.721–0.910), with 65.1% sensitivity and 88.6% specificity at a cutoff of 0.344. CDFI and SMF yielded AUCs of 0.708 and 0.755, with corresponding sensitivities of 74.3% and 77.1% and specificities of 62.8% and 65.1%, respectively (**Figure 7**).

**Figure 8:**
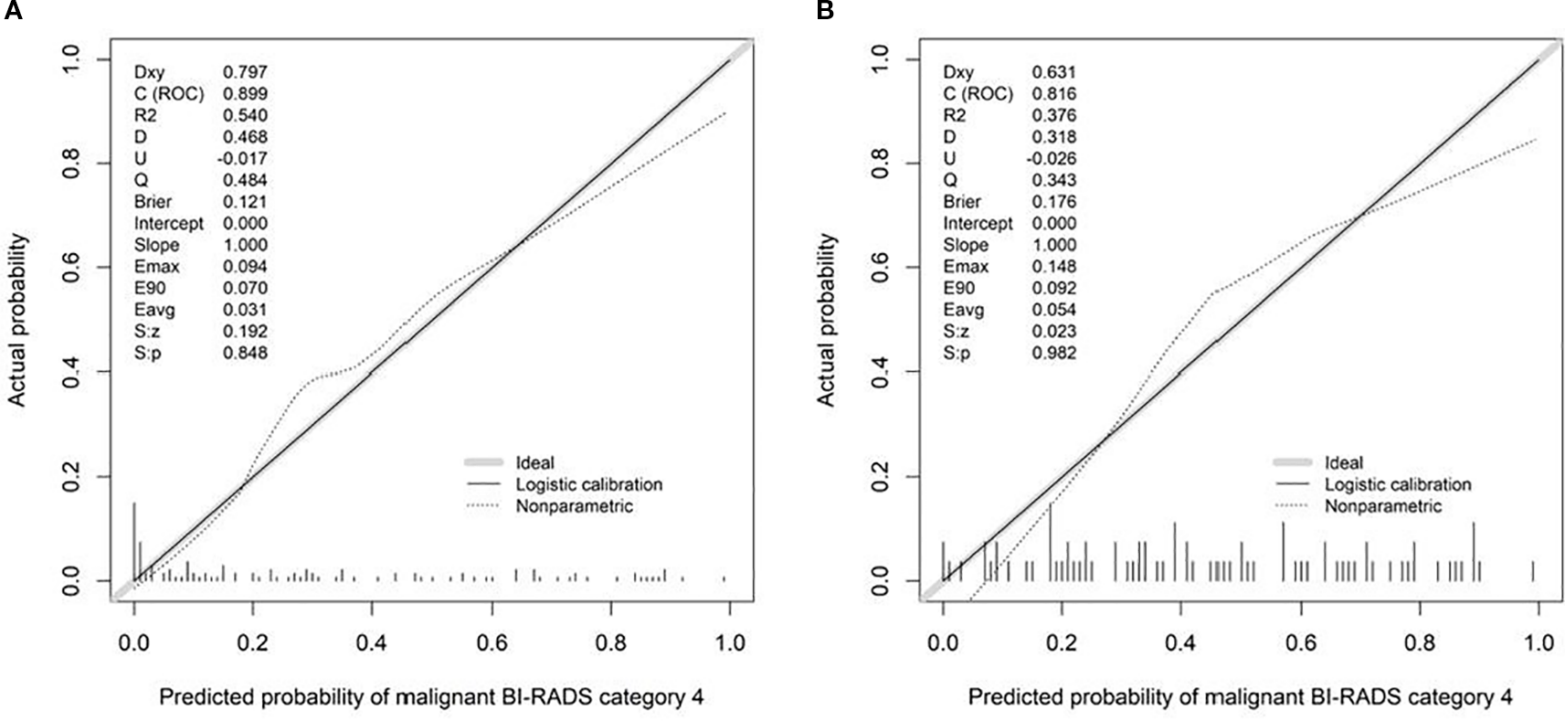
Calibration curves of Model 1-2 (**A**: Model 1, **B**: Model 2).

**Figure 9:**
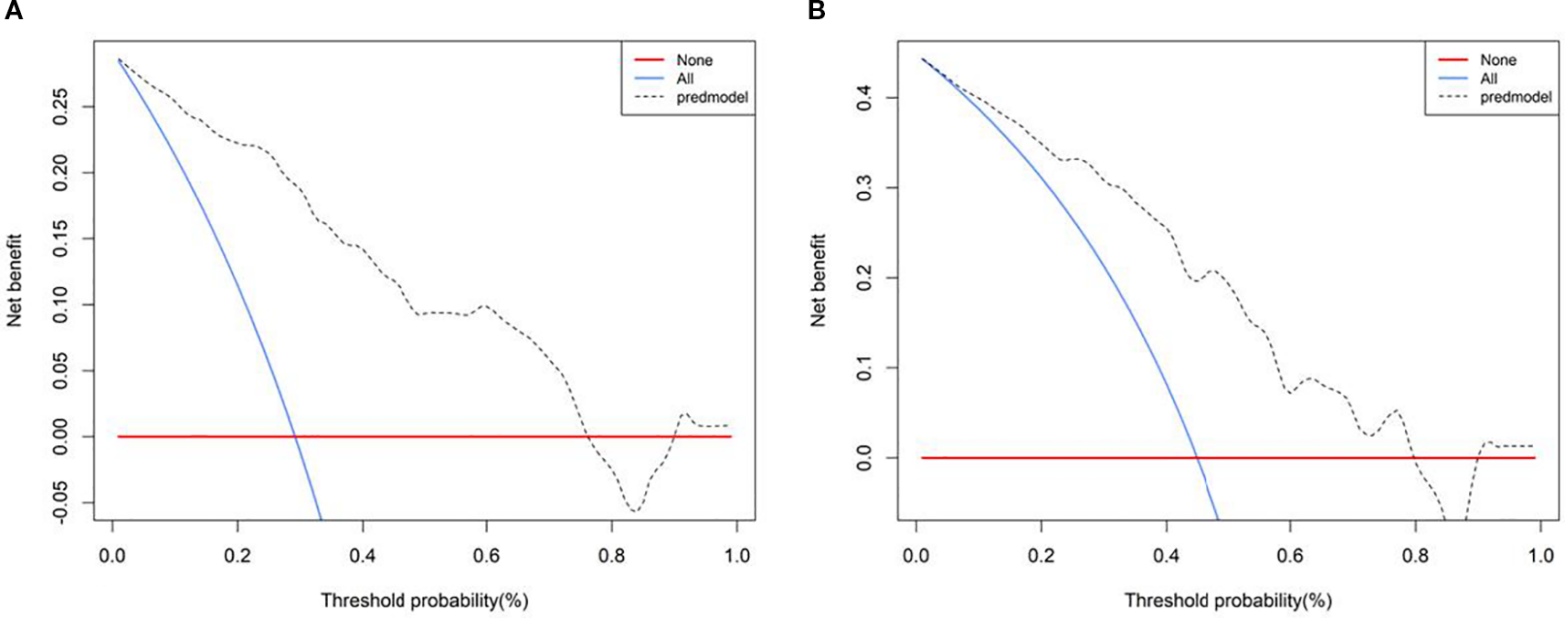
Decision curve of Model 1-2 (**A**: Model 1, **B**: Model 2).

Calibration curves demonstrated good agreement between predicted and observed outcomes, which was supported by non-significant Hosmer–Lemeshow test results (*P* > 0.05; **Figure 8**). Decision curve analysis confirmed the clinical utility of both SRUS models across threshold probabilities of 0–0.77 (malignant vs. benign) and 0–0.79 (malignant vs. hypervascular benign; **Figure 9**).

## 4 Discussion

Neovascularization, a hallmark of cancer, plays a pivotal role in tumor progression and metastasis (13). Microvessel density (MVD), a key quantitative marker of angiogenesis in solid tumors, is not only a risk factor for breast cancer metastasis but also a predictor of poor prognosis (14). However, MVD assessment relies on postoperative immunohistochemical analysis of excised specimens, precluding real-time preoperative evaluation. Furthermore, conventional color Doppler flow imaging (CDFI) is hindered by Nyquist limit effects, exhibiting low sensitivity for microvessels <1 mm in diameter and slow blood flow (<5 cm/s). Additionally, it is affected by noise susceptibility and angle dependence (15). While CEUS improves microcirculation visualization via microbubble tracing (16), it remains restricted by the diffraction limit, impairing its ability to resolve microvascular architecture. These technical shortcomings highlight the need for advanced high-resolution vascular imaging. Currently, no non-invasive imaging modality enables real-time visualization of microvascular characteristics in clinical practice. SRUS surpasses the diffraction limit by localizing, tracking, and reconstructing microbubble trajectories, thereby achieving micrometer-scale microvascular imaging and parametric analysis. This technique enables the visualization of vessels as small as 20 μm, representing a >10-fold resolution enhancement over conventional methods and facilitating the early detection of microvascular alterations. Given its exceptional resolution, SRUS holds significant promise for investigating microvascular dynamics in breast lesions; however, its feasibility and clinical utility in breast pathology remain unexplored.

Certain hypervascular benign lesions, such as intraductal papilloma, hypervascular inflammatory lesions, adenoma, and hyperplasia, exhibit enhancement patterns on CEUS that overlap with those of malignant tumors due to their active cellular proliferation or inflammatory characteristics. This overlap also complicates accurate diagnosis (17). To address this, the present study stratified benign breast nodules into hypovascular and hypervascular subgroups. We employed SRUS to differentiate benign from malignant BI-RADS category 4 lesions and systematically evaluated its diagnostic efficacy in distinguishing malignant tumors from hypervascular benign lesions.

Felix et al. (18) quantified MVD by counting CD31(+) vessels in breast biopsy specimens, demonstrating significantly higher MVD in ductal carcinoma *in situ* and invasive breast cancer tissues compared to benign lesions. Additional studies (14) have corroborated that malignant breast lesions exhibit elevated microvascular density relative to benign counterparts. In this study, statistically significant differences (all *P* < 0.05) were observed in density (max, mean), velocity (max, mean), and perfusion index between malignant and benign groups. These parameters were further elevated in both malignant and hypervascular benign subgroups compared to hypovascular benign lesions (all *P* < 0.001), likely reflecting the dense vascular networks and high arteriovenous shunt rates characteristic of malignant tumors. Malignant neovascularization progresses centripetally, forming branched vascular networks that infiltrate tumor subregions and abruptly increasing regional and total blood flow. Simultaneously, structural deficiencies in malignant vasculature—including smooth muscle layer loss, impaired vasomotor function, and tumor thrombus formation—promote arteriovenous shunting, accelerating flow velocity and elevating perfusion (19). However, no statistically significant differences (*P* > 0.05) were detected between malignant and hypervascular benign groups, likely due to overlapping pathophysiological mechanisms. Hypervascular benign lesions, such as complex fibroadenomas, exhibit localized proliferative activity and atypical ductal hyperplasia, while inflammatory lesions undergo capillary remodeling driven by IL-8, TNF-α, and other cytokines involved in tissue repair and leukocyte infiltration (20). These shared processes contribute to similar sonographic hemodynamic patterns in hypervascular benign and malignant lesions, a key factor in imaging misdiagnoses.

The vessel ratio, which quantifies vascular abundance, showed progressively higher values from hypovascular benign to hypervascular benign to malignant subgroups, with the differences being statistically significant (*P* < 0.05). However, the considerable standard deviations observed, especially within the malignant and hypervascular benign groups, indicated a significant overlap in values between these two categories. This overlap precluded the establishment of a reliable discriminatory threshold for clinical diagnosis, explaining why vessel ratio was not an independent discriminator between malignant and hypervascular benign lesions in our multivariate analysis.

Prior studies (21) using murine models of human colorectal cancer (CRC) xenografts demonstrated that initial microvascular complexity was approximates 1.7, stabilizing at 1.84 due to tumor growth-induced vascular remodeling. During tumor regression, pathological vascular networks exhibit reduced complexity, reaching levels between healthy tissues and growing tumors. Consistently, our findings revealed a statistically significant progressive increase in complexity with breast lesion progression: malignant lesions (1.63 ± 0.11) > hypervascular benign (1.44 ± 0.24) > hypovascular benign (1.16 ± 0.26) (all *P* < 0.001). These morphological and functional vascular disparities suggest that penetrating vessels, branching patterns, and vascular disorganization may serve as malignancy indicators (22). Furthermore, our results demonstrated significantly higher curvature (A/E) in malignant lesions (1.44 ± 0.45) compared to hypovascular (1.11 ± 0.13) and hypervascular benign subgroups (1.19 ± 0.18). This phenomenon likely reflects tumor hypoxia and acidosis, driven by inadequate oxygen supply and impaired metabolic waste clearance during rapid proliferation. Hypoxia, a key driver of tumor neovascularization, fosters the development of tortuous and disorganized vascular networks.

This study identified maximum velocity, complexity level, and curvature (A/E) as independent predictors for differentiating benign from malignant BI-RADS category 4 breast lesions. Complexity level and curvature (A/E) further served as discriminators between malignant and hypervascular benign lesions. The corresponding nomogram models demonstrated strong diagnostic performance: the model for benign-malignant differentiation achieved an AUC of 0.899 with 94.3% sensitivity and 76.5% specificity, while the model for malignant versus hypervascular benign lesions attained an AUC of 0.816 with 88.6% sensitivity and 65.1% specificity. Both models exhibited excellent calibration and clinical utility in decision curve analysis, supporting SRUS as a robust tool for breast lesion characterization.

Superb Microvascular Imaging (SMI), also referred to as SMF, improves microvascular visualization compared to CDFI by preserving low-velocity flow signals while suppressing motion artifacts. In our cohort, both CDFI and SMF showed moderate efficacy in discriminating benign from malignant lesions, with AUCs of 0.707 and 0.771, respectively. SRUS significantly outperformed both, achieving an AUC of 0.899—driven by high sensitivity (94.3%) without compromising specificity (76.5%). More importantly, it is a clinically challenging task to distinguish malignant from hypervascular benign lesions, as CDFI and SMF performed poorly (AUCs: 0.708 and 0.755, respectively); however, in distinguishing between these lesions, the SRUS-based model achieved an AUC of 0.816 with high specificity (88.6%), highlighting its potential to reduce unnecessary biopsies.

A large meta-analysis reported pooled sensitivity and specificity of CEUS as 0.87 and 0.79, respectively (23). In comparison, in this study, SRUS demonstrated superior sensitivity (94.3%) with comparable specificity (76.5%). This high sensitivity is critical for reliably ruling out malignancy. By providing a quantitative assessment of microvascular architecture, SRUS enables a more objective and precise diagnostic approach. Its superior performance, particularly in challenging cases, such as hypervascular benign lesions, strongly supports its integration into clinical practice.

This study has several limitations. First, it was a single-center study with a relatively limited number of malignant lesions (n=35), which may affect the generalizability of our findings. Additionally, the maximum diameter of malignant lesions was significantly larger than that of benign ones. We acknowledge that larger tumor size could theoretically influence microvascular abundance. However, the SRUS-derived parameters of vascular complexity and curvature (A/E) were identified as independent predictors of malignancy in the multivariate logistic regression analysis that included and adjusted for lesion size. This strongly suggests that these microarchitectural features capture the intrinsic hallmarks of malignant angiogenesis beyond a mere size-effect. Nevertheless, future multicenter studies with a larger, size-matched cohort are warranted to further validate our findings and explore the diagnostic value of SRUS across different tumor sizes.

## 5 Conclusion

In summary, SRUS is a novel hemodynamic visualization technique that quantifies micron-level vascular parameters with high precision. This study innovatively employed SRUS to quantitatively assess the microvascular characteristics of breast tumors, offering valuable insights for noninvasive evaluation of tumor vasculature. Preliminary findings highlight the clinical significance of SRUS parameters in differentiating benign from malignant BI-RADS category 4 lesions, with complexity level and curvature (A/E) demonstrating superior discriminatory power in distinguishing malignant tumors from hypervascular benign lesions. As a pioneering exploration in ultrasound microvascular imaging, this research establishes a new technical framework for optimizing clinical decision-making in breast tumor management and provides a critical reference for advancing functional ultrasound applications.

## Data Availability

The original contributions presented in the study are included in the article/supplementary material. Further inquiries can be directed to the corresponding author.
